# Wild deer as potential vectors of anthelmintic-resistant abomasal nematodes between cattle and sheep farms

**DOI:** 10.1098/rspb.2013.2985

**Published:** 2014-04-07

**Authors:** C. Chintoan-Uta, E. R. Morgan, P. J. Skuce, G. C. Coles

**Affiliations:** 1 School of Veterinary Sciences, University of Bristol, Bristol BS40 5DU, UK; 2 Moredun Research Institute, Penicuik EH26 0PZ, UK

**Keywords:** nematodes, livestock, deer, anthelmintics, anthelmintic resistance, *Haemonchus contortus*

## Abstract

Gastrointestinal (GI) nematodes are among the most important causes of production loss in farmed ruminants, and anthelmintic resistance is emerging globally. We hypothesized that wild deer could potentially act as reservoirs of anthelmintic-resistant GI nematodes between livestock farms. Adult abomasal nematodes and faecal samples were collected from fallow (*n* = 24), red (*n* = 14) and roe deer (*n* = 10) from venison farms and areas of extensive or intensive livestock farming. Principal components analysis of abomasal nematode species composition revealed differences between wild roe deer grazing in the areas of intensive livestock farming, and fallow and red deer in all environments. Alleles for benzimidazole (BZ) resistance were identified in *β-tubulin* of *Haemonchus contortus* of roe deer and phenotypic resistance confirmed *in vitro* by an egg hatch test (EC_50_ = 0.149 µg ml^−1^ ± 0.13 µg ml^−1^) on *H. contortus* eggs from experimentally infected sheep. This BZ-resistant *H. contortus* isolate also infected a calf experimentally. We present the first account of *in vitro* BZ resistance in wild roe deer, but further experiments should firmly establish the presence of phenotypic BZ resistance *in vivo.* Comprehensive in-field studies should assess whether nematode cross-transmission between deer and livestock occurs and contributes, in any way, to the development of resistance on livestock farms.

## 1. Introduction

It is well documented that the economics of cattle and sheep farming are negatively affected by high burdens of gastrointestinal (GI) nematodes through decreases in meat and milk productivity and reproduction [1–3]. Effects such as reduced feed conversion efficiency [4,5] and milk production [6] in cattle have been demonstrated and quantified at farm level. In sheep, studies of the economic impact of nematode infection are limited, but Nieuwhof & Bishop [7] estimated that in the UK GI parasites account for up to £84 million in annual losses, the main costs being owing to a reduction in the growth rate of lambs, and the cost of the treatment and control programmes.

Some of the nematode species infecting livestock mainly parasitize a single host, being found in other hosts only on very rare occasions—these are categorized as specialist species. On the other hand, some species are commonly found in more than one host, and these species are categorized as generalist. Recent molecular DNA evidence suggests that the Ostertagiinae have evolved in close relation with bovids and cervids [8], and nematode species strongly associated with each host species have evolved. By contrast, the Haemonchinae appear as generalist species, and it has been demonstrated that, under experimental conditions, *Haemonchus contortus* is able to be passed between cattle, sheep and white-tailed deer, and back to cattle and sheep from deer [9]. More recently, molecular genetic analysis investigating the divergence of the internal transcribed spacer (ITS) and mitochondrial gene sequences has supported this finding by suggesting that sheep, wild bovids and wild deer in northern Italy share a common field population of *H. contortus* [10].

Less is known about transmission to deer of other species commonly found in cattle and sheep, but nematodes thought to be specific to cattle and sheep were found in wild deer in a number of surveys, although no direct experimental evidence for cross-transmission exists. Pato *et al.* [11] concluded that there was cross-transmission of GI nematodes between roe deer and cattle in Spain as they identified eggs of *Nematodirus* spp., *Trichuris* spp. and *Capillaria* spp. in the faeces of both roe deer and cattle. Their conclusion was further supported by examination of adult GI burdens, which revealed that *Ostertagia* (*Teladorsagia*) *circumcincta, Trichostrongylus axei* and *Cooperia punctata* were found in both cattle and roe deer. Furthermore, Pato *et al.* [12] also demonstrated that roe deer in the northwest of the Iberian Peninsula were widely and intensely infected with GI nematodes that are considered specific to domestic ruminants (*Ostertagia*, *Nematodirus*, *Trichostrongylus*, *Teladorsagia*, *Chabertia*, *Cooperia*, *Haemonchus*), and that they may act as potential reservoirs of nematodes for domestic ruminants.

While the cross-transmission of GI nematodes between wild deer and livestock is implied from the studies above, to date, there has been no investigation into whether deer could act as potential vectors of anthelmintic-resistant nematodes between cattle and sheep farms. Owing to the high potential of these GI nematodes to cause economic losses, commercial livestock production has been supported by heavy reliance upon anthelmintic drugs, without taking into account methods that minimize the development of anthelmintic resistance [13]. Such practices facilitated the selection of anthelmintic-resistant nematodes in both sheep and cattle, which has now been widely reported globally. While New Zealand is one of the countries that has well documented its extent [14,15], in the UK, anthelmintic-resistance reports are emerging, with triple class resistance being reported in sheep [16,17] and widespread inefficacy of macrocyclic lactones (ML) in cattle (K. Stafford & G.C.C. 2012, unpublished data).

This study aimed to investigate transmission of abomasal parasitic nematodes between cattle, sheep and wild deer in the UK, and to determine whether anthelmintic-resistant nematodes are present in the wild deer population. Given the lack of treatment in wild deer, this would indicate nematode transmission from livestock to deer and raise the possibility that deer can transfer anthelmintic resistance between livestock farms. Abomasal nematodes were selected for study because they are economically the most important parasites of livestock, were previously shown to overlap in species composition with those of deer and include the species of greatest current concern with regard to anthelmintic resistance. The abomasum is also by far the most important site of colonization within the GI tract and has been shown to contain a higher number of nematode species than the large or small intestines individually [12].

## 2. Material and methods

### (a) Animals

A total of 48 samples were collected from fallow (*n* = 24), red (*n* = 14) and roe deer (*n* = 10) grazing in three types of environment: farmed deer (fenced off from livestock or wildlife; fallow: 10; red: 8), wild deer grazing in the areas of extensive cattle farming (fallow: 4; red: 3) and wild deer grazing in the areas of intensive cattle farming (fallow: 10; red: 3; roe: 10). Electronic supplementary material SI further describes the samples collected mentioning location, the species of deer collected, the number of each species collected and the type of environment. Deer were killed by rifle, and none of the deer sampled had damage to the abdomen. Deer collected from venison farms were not treated with anthelmintics. It was not possible to collect farmed roe deer, and culling of roe deer was not practised in the area of extensive farming sampled (New Forest, UK).

### (b) Sample processing

Samples collected from each deer included faecal samples and the abomasum. Faecal samples were used for a faecal egg count (FEC) and for extraction of nematode eggs. The abomasum was used for enumeration and description of the abomasal nematode burden. Each of these techniques is described below.

#### (i) Faecal egg counts

FECs were performed using the FLOTAC apparatus, described by Cringoli [18] and validated for use in red deer by Bauer *et al.* [19]. Ten gram samples of faeces were homogenized in 90 ml of water by shaking by hand, and the nematode eggs concentrated by centrifugation at 405*g* for 2 min. The supernatant was decanted, and the eggs resuspended in 10 ml of saturated sodium chloride solution. Five millilitre of the suspension was added to one of the chambers of the FLOTAC apparatus. One chamber per animal was counted, giving a detection limit of two eggs per gram (epg). The FLOTAC apparatus was centrifuged at 67*g* for 5 min to separate the eggs from the debris. The eggs were counted in the entire cell at 40 times magnification, using a Cobra (Vision Engineering, UK) microscope. Average FECs were calculated for each species of deer and 95% confidence limits determined by bootstrapping [20] over 200 iterations, as parasite burdens do not follow the normal distribution, using SPSS (IBM, USA). FECs for the faecal egg count reduction tests (FECRTs) were conducted using the standard modified McMaster method, with detection limit 50 epg, which was considered adequate in the light of high starting counts.

#### (ii) Adult abomasal nematode burden estimation

Following the processing of the abomasal contents according to the method described in MAFF [21], an aliquot of 10% of the contents, by weight, was examined under a microscope (Vision Engineering, Cobra Stereo Zoom), under six times magnification, for the presence of adult nematodes. Females and males were counted and stored separately in 70% v/v ethanol in water. The total adult abomasal nematode burden was estimated by summing the number of females and males, and multiplying by 10. Average adult abomasal nematode burdens were calculated for each species of deer, with confidence limits calculated as for the FEC above.

#### (iii) Nematode identification

Only males were identified visually, according to spicule morphology, using the key described by Skrjiabin *et al.* [22]. A maximum of 40 males were identified from each sample. In those samples that had more than 40 males, 40 were chosen by spreading male nematodes in a Petri dish by shaking. Then, using a 5 × 5 grid and a random number generator, all males within individual blocks as determined by the random number generator were collected until a number of 40 was reached. The species composition of the total abomasal nematode burden was estimated from this count. Visual identification of eight male nematodes was not possible owing to the similar spicule morphology between *O. ostertagi* and *O. leptospicularis*, and these were identified using molecular techniques. DNA was extracted from these nematodes using the DNeasy blood and tissue kit (Qiagen, UK), according to the manufacturer's instructions. A region of the internal transcribed spacer 2 (ITS2) was amplified using the primers detailed for *O. ostertagi* by Zarlenga *et al.* [23]. The amplicon was sequenced through dideoxy sequencing, and fixed nucleotide differences [24] were used to distinguish between the two species as the ITS2 region has lower intraspecific than interspecific variation in nematodes [25]. The following two fixed nucleotide differences were used: at position 100, A indicated *O. ostertagi* and G indicated *O. leptospicularis*; at position 111, T indicated *O. ostertagi*, and A followed by an insertion of TG indicated *O. leptospicularis*. The PCR was validated on known *O. ostertagi* provided by the Moredun Research Institute (Edinburgh, UK).

#### (iv) Nematode egg extraction

The salt flotation method described by the Ministry of Agriculture, Fisheries and Food [21] was used. Faecal samples were homogenized in water, and the resulting mixture centrifuged at 405*g* for 2 min. The supernatant was decanted and the pellet re-suspended in saturated sodium chloride. A coverslip was placed over the tube, ensuring a tight seal with no air in the tube, and the tubes centrifuged at 67*g* for 5 min. The coverslip was washed into a 14 ml Falcon tube, the eggs counted in five 10 µl aliquots and the average number of eggs per 10 µl calculated. The volume of water was adjusted to give approximately 100 eggs per 10 µl.

### (c) Nematode cross-transmission between wild cervids and domestic livestock

#### (i) Abomasal nematode species diversity

Nematode species diversity was summarized in each species of deer by calculating the total number of nematode species found in each host species, and the mean number of nematode species found per individual deer.

#### (ii) Statistical analysis

Principal component analysis (PCA) was undertaken on nematode counts from individual deer, considering major and minor morphs as a single species. Zero values were entered for parasite species absent in individual deer. Scores for individual deer were calculated for the first two principal components and these were used to graphically represent the data, following standardization around zero on each axis. The effect of the species of deer and the environment was further investigated using a generalized linear model (GLM) on the values of the principal components for individual deer. Paired *t*-tests were used to study the influence of the environment on the nematode fauna of fallow and red deer, with Bonferroni correction applied to the critical *p*-value in order to take account of multiple comparisons. SPSS was used for the aforementioned tests.

#### (iii) *In vivo* cross-infection

To confirm cross-infection of nematode species, an *in vivo* cross-infection study was undertaken. Approximately 3000 infective third-stage larvae were cultured from bulked faeces from the roe deer sampled in this study. Abomasal parasites in the roe deer were identified before the experimental infection, but no species identification of the larvae was undertaken as it was intended to assess which of the species of nematodes occurring in the natural population in deer would be able to infect the livestock. Recovered larvae were used to infect a single calf, using a trickle infection with a quarter of the larvae (approx. 750 larvae) given as a single daily dose over four consecutive days. This method of infection was used as it has been observed that infection with a lower number of larvae over consecutive days results in a more stable and reliable infection compared with a single high dose, and mimics natural infection more closely. After 21 days, faeces from the calf were collected and used for larval culture and extraction. Approximately 8000 larvae were obtained and they were used to infect a single lamb, again using a trickle infection. Faeces from this lamb were collected for two weeks starting from day 21 and used for larval culture and extraction. The calf and the lamb were demonstrated as parasite-free by FECs using the FLOTAC method before experimental infection. No anthelmintic treatment was given pre-infection to either the calf or the lamb. Both the calf and the lamb were slaughtered at the end of the study and abomasal nematodes were collected from the calf.

### (d) Benzimidazole resistance testing

#### (i) Molecular tests

All *H. contortus* individuals collected from a 10% aliquot of abomasal nematodes from two wild roe deer were tested for benzimidazole (BZ) resistance using the PCR detailed by Coles *et al.* [26]. The forward primer GGAACGATGGACTCCTTTCG and the reverse primer GGGAATCGAAGGCAGGTCGT were used to amplify a 750 bp product from the isotype-1 β-tubulin gene. The NovaTaq hot start master mix was used, and PCR cycling conditions were as follows: 15 min at 95°C for activation of the DNA polymerase, 39 cycles of 30 s at 94°C, 90 s at 60°C, 2 min at 72°C, final extension of 10 min at 72°C. The PCRs were checked by agarose gel electrophoresis and gel purified using the gel purification kit (Qiagen, UK). The purified amplicons were sent for sequencing to Dundee DNA and Sequencing Services, UK.

#### (ii) *In vitro* tests: the egg hatch test

To confirm the results of the molecular tests for BZ resistance, faeces from the lamb mentioned in the *in vivo* cross-transmission study above were used to extract nematode eggs. This was done as described above, and the eggs were used in an egg hatch test (EHT). The EHT was carried out according to the method described by von Samson-Himmelstjerna [27]. Briefly, the eggs were incubated in increasing concentrations of thiabendazole diluted in DMSO in triplicate, in 24-well plates, for 48 h, at 25°C. At the end of the incubation period, the total number of eggs and the number of eggs hatched in each well was counted. The probit function in SPSS was used to calculate EC_50_ for the test. A discriminating dose (LD_99_) of 0.1 µg ml^−1^ thiabendazole was also used as advocated by Cudekova *et al.* [28].

#### (iii) *In vivo* tests: the faecal egg count reduction test

To confirm phenotypic BZ resistance *in vivo* and to investigate ML resistance, the larvae extracted from the single lamb above were used to infect six other lambs. All lambs were confirmed as nematode-free by FECs using the FLOTAC method. Each of the lambs was given approximately 5000 larvae as a trickle infection, over 4 days with a quarter of the dose each day. At 21 days, a FEC was done on each of the lambs. The lambs were weighed and treated orally as follows: two control lambs with sterile saline, two lambs treated with 5 mg kg^−1^ fenbendazole (full therapeutic dose) and two lambs treated with half the manufacturer's recommended dose of ivermectin: 0.1 mg kg^−1^. This dose was used because previous trials have indicated that ivermectin has a high overkill [29], and that the half-dose can therefore give an early indication of developing resistance. After 14 days, all six lambs were euthanized, and the abomasum and intestines collected for parasitological examination as described above. FECs were also performed in each lamb at this point.

## 3. Results

### (a) Abomasal nematode burdens in deer, and cross-transmission to livestock

#### (i) Nematode burdens and faecal egg counts

**Figure 1.**
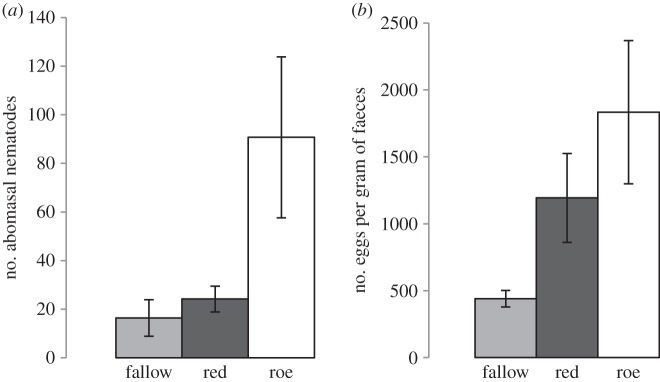
The mean (*a*) abomasal nematode burden and (*b*) FEC of fallow, red and roe deer. Confidence intervals (95%) are shown as bars and were calculated using bootstrapping (200 iterations). The three means are statistically different—no mean of one group is included in the 95% CI of other groups.

Abomasal nematode burdens were determined by direct abomasal counts and indicated that roe deer had the highest burdens, followed by red and then fallow deer (figure 1*a*). FECs were determined in order to gain an indication of pasture contamination potential rather than a measure of abomasal nematode burden. Again, roe deer had the highest counts, followed by red, then fallow deer. Mean nematode FECs for the three species of deer studies are given in figure 1*b*.

#### (ii) Nematode species identified

All three nematode species commonly found in fallow (*Ostertagia asymmetrica*), red (*Spiculopteragia spiculoptera*) and roe deer (*Ostertagia leptospicularis*), respectively, were identified in this study and species considered livestock-specific were also identified. The prevalence and mean abundance of all nematode species identified in the abomasum of each host species is given in table 1. The presence of *T. colubriformis* in the abomasum, which is usually an intestinal parasite, could be explained by leakage of intestinal contents into the abomasum between shooting and sample collection. The number of abomasal nematodes of each species found within individual deer is given in electronic supplementary material SII.

**Table 1.** The prevalence and mean abundance of nematode species identified in the abomasum of each host species sampled. The species described are all the nematode species identified in each deer species.

nematode species	fallow (*n*=24)		red (*n*=14)		roe (*n*=10)	
	prevalence (%)	mean abundance (range)	prevalence (%)	mean abundance (range)	prevalence (%)	mean abundance (range)
*Haemonchus contortus*	not identified		not identified		20	7 (70 in a single deer)
*Ostertagia assymetrica*	96	119.6 (0–280)	93	111.4 (0–280)	not identified	
*Ostertagia leptospicularis*	71	49.6 (0–170)	64	27.1 (0–100)	100	211 (70–300)
*Ostertagia ostertagi*	not identified		not identified		70	10 (0–20)
*Spiculopteragia spiculoptera*	42	14 (0–30)	100	143.5 (10–470)	100	78 (10–210)
*Trichostrongylus axei*	8	1.7 (0–30)	7	0.7 (0–10)	80	60 (0–270)
*Trichostrongylus colubriformis*	not identified		not identified		70	16 (0–40)

#### (iii) Species diversity

Roe deer had the highest abomasal nematode species diversity, followed by red and fallow deer, as indicated by the total and mean number of nematode species identified in each species of deer: in fallow deer 3 (mean, *x* = 2.1 ± s.d. 0.49) species were observed, in red deer 4 (*x* = 2.6 ± 0.62) species and in roe deer 6 (*x* = 4.3 ± 1.05) species. The median number of species was significantly different across the three host species (Kruskal–Wallis test, chi-squared test: 21.99, 3 d.f., *p* < 0.0001).

#### (iv) Differences in abomasal nematode fauna between deer species

**Figure 2.**
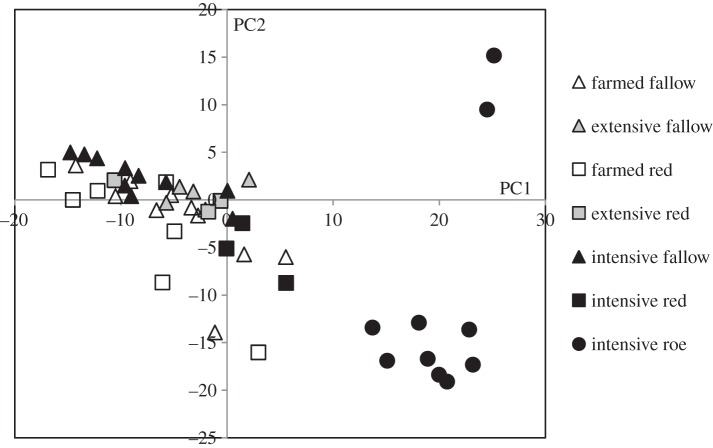
Principal component analysis of abomasal nematodes in each deer species and type of habitat sampled. Roe deer from intensive farming areas cluster at values above 10 of principal component (PC) 1, whereas fallow and red deer cluster at values under 10 of PC1. Kaiser–Meyer–Olkin measure of sample adequacy = 0.679. Bartlett's test of sphericity chi-square = 111.27, *p* < 0.0001. Unstandardized component loadings (PC1, PC2, respectively) are *H. contortus* 0.906, 0.172; *O. assymetrica* 0.703, −0.703; *O. leptospicularis* 0.701, 0.701; *O. ostertagi* 0.625, −0.538; *S. spiculoptera* 0.524, 0.620; *T. axei* 0.057, −0.348; *T. colubriformis* −0.642, 0.244. The proportion of total variance explained by PC1 and 2, respectively, was 44.5% and 24.3%.

Having identified the species of nematode present in each of the deer species studied, PCA was used to assess the differences in abomasal nematode fauna of deer. This analysis apportioned variation in the presence and abundance of different parasites at individual level, integrating information on all nematode species, and complements the host species-level data in table 1. Roe deer grazing in the areas of intensive farming had an abomasal nematode fauna different from that of fallow and red deer, irrespective of where the latter two were grazing (figure 2). In the PCA, roe deer were consistently separated along PC1 with high positive values compared with other groups, and generally negative on PC2 (with two exceptions). The factor loadings suggest that this is explained by greater general abundance of parasites in roe deer compared with the other deer groups, including livestock-associated species such as *O. ostertagi, Trichostrongylus colubriformis*, *T. axei* and *H. contortus*.

#### (v) Influences on abomasal nematode fauna

The unbalanced number of samples across grazing environments and deer species confounds the data, and conclusions on the contribution of factors to the determination of abomasal nematode fauna should be interpreted with this limitation in mind. However, a GLM (*F* = 33.875; *p* < 0.001) on the first principal component (PC1) of the PCA indicated that deer species (*F* = 53.34; *p* < 0.001) was a significant factor explaining the abomasal nematode fauna. The environment also played a role, but only in interplay with species of deer (*F* = 5.161; *p* = 0.01) and not alone (*F* = 1.23; *p* = 0.30). Tukey's *post hoc* analysis of the GLM highlighted that the roe deer sampled had a different abomasal nematode fauna to fallow (*p* < 0.001) and red deer (*p* < 0.001). A second PCA restricted to the small numbers of deer sampled from the areas of intensive livestock grazing also showed separation of roe from red and fallow deer (results not shown), confirming that within this environment, deer species was a major determinant of abomasal nematode fauna.

Only red and fallow deer were sampled from all three types of environment, and paired *t*-test analysis of the influence of grazing environment within each of these species showed no differences between the three types of environment (*p* > 0.21, critical *p* with Bonferroni correction = 0.016). Together with the GLM result above, this indicates that nematode fauna was little affected by the environment alone in red and fallow deer. The result of the GLM indicating that the environment was an influencing factor in interplay with deer species could be a result of sampling bias and the fact that roe deer were only sampled in the intensive environment.

#### (vi) *In vivo* cross-transmission

Larvae extracted from cultures of roe deer faeces successfully established an infection in the experimental calf. Between days 21 and 35 post-infection, the FECs ranged between 3 and 8 epg. A total of 19 nematodes were recovered from two 10% aliquots of abomasal washes—12 females and 7 males. Females were not identified, and of the seven males collected one was *H. contortus* (confirmed using the discriminant function of Jacquiet *et al.* [30], one was *S. spiculoptera* and the rest were *O. leptospicularis*. An abomasal nematode burden of 95 was estimated from two aliquots of 10%.

The single lamb that was given larvae extracted from faecal cultures from the experimental calf was successfully infected. FECs fluctuated between 1500 and 3000 epg throughout the period between 21 and 35 days post-infection. Of the males recovered from a 10% aliquot, 70 of 76 were *H. contortus*, the remainder being *O. leptospicularis.* A total abomasal nematode burden of 1650 was estimated.

### (b) Anthelmintic resistance

#### (i) Molecular evidence

Twenty-one individual *H. contortus*, isolated from wild roe deer, were genotyped for the F167Y, E198A and F200Y polymorphisms in the *isotype-1 *β*-tubulin* gene in order to study BZ resistance. This indicated that the isolate was resistant to BZ and the frequencies of the resistant alleles at each genetic locus were: 64.5% at position (P) 200, 0% at P198 and 7% at P167. The frequencies of resistant alleles identified in three individuals of *H. contortus* recovered from the calf infected with nematode eggs from roe deer were 33.3% at P167, 0% at P198 and 33.3% at P200. In five individuals of *H. contortus* sequenced from the lamb infected with larvae resulting from the experimental infection of the calf, resistant alleles were only identified at P200 (60% resistant allele frequency). The results highlight that the anthelmintic-resistant nematodes from roe deer were able to successfully infect the experimental calf and lamb. Sequencing data from the abovementioned *H. contortus* individuals were deposited to GenBank with the following accession numbers: KJ018259–KJ018261 for the three individuals recovered from the experimental calf, KJ018262–KJ018266 for the five individuals recovered from the experimental lamb and KJ018267–KJ018287 for the individuals isolated from wild roe deer.

#### (ii) *In vitro* evidence

Analysis of the results of the EHT revealed a half-maximal effective concentration (EC_50_) of 0.149 µg ml^−1^ thiabendazole with a confidence interval of 0.136–0.162 (the raw data from this test are included in electronic supplementary material SIII). An EC_50_ over 0.1 µg ml^−1^ is indicative of resistance to benzimidazoles. The percentage of eggs surviving at 0.1 µg ml^−1^ thiabendazole was 62.43%, which is similar to the resistant allele frequency at P200 given above.

#### (iii) *In vivo* evidence

The results of the FEC reduction test are given as electronic supplementary material SIV. Although the average efficacy of treatment was 91.5%, suggesting the presence of phenotypic resistance to BZ in the *H. contortus* isolate from wild roe deer, no firm conclusions can be drawn owing to a number of confounding factors, which are discussed below. There was no indication of resistance to MLs even after treatment with half the manufacturer's recommended dose of ivermectin (100% efficacy of treatment in both lambs).

## 4. Discussion

A small number of studies undertaken on wild deer in the UK over 40 years ago demonstrated the presence of cattle and sheep nematodes in these wild animals [31,32], but no further studies have been published, and none since anthelmintic resistance was highlighted as an emerging issue. This study represents the largest survey of abomasal nematodes in wild deer undertaken in the UK in the past decade, and the only one anywhere to also assess anthelmintic efficacy against the nematodes recovered.

The data established that roe deer tended to have higher abomasal nematode burdens and FEC than fallow or red deer, even when grazing in the same geographical areas. Approximately half of the nematodes present in individual roe deer were generalist species, in contrast to red and fallow deer, in which species associated with cervids were dominant. Roe deer are the most numerous and the most widespread species of deer in Britain, and commonly graze pastures used by livestock. Our analysis was unable to distinguish fully between the effects of host and environment on nematode fauna, because all roe deer were sampled in the areas intensively used by livestock. This was inevitable, because roe deer are not farmed, and were not culled in the extensively grazed area from which samples were collected. However, the lack of differences in the nematode fauna of fallow and red deer grazing in different environments, including intensively grazed farmland, suggests that roe deer are particularly susceptible to livestock-associated nematode species, or that they have greater opportunity to encounter them. The grazing environment could still be a factor influencing abomasal nematode fauna, but in order to firmly determine its influence a further study, with a balanced sampling design (including roe deer that are farmed and those grazing in extensively managed areas) would be necessary; but this is unlikely to be possible given the above constraints. Parasite transmission between livestock and wildlife in both directions is influenced by complex interactions between habitat use and climate [33,34], and greater understanding of the grazing patterns of deer on farmland is needed to predict patterns of cross-infection. Studies of the population dynamics of roe deer when kept at high stocking density showed high susceptibility to parasite infection [12,35]. Other studies have shown that roe deer change their habitat selection [36] and diet [37] in fragmented agricultural habitats, making use of grass on livestock pastures, and this would increase their exposure to livestock parasites. Increasing deer population density and habitat fragmentation caused by modern farming practices could have led roe deer to increasingly graze rather than browse, and to consequently become exposed to higher infection pressure from GI nematodes. Separately, the risk of other parasitic diseases in deer has been shown to increase with landscape fragmentation [12,38].

Insights into the field population of abomasal nematodes in wild deer grazing in other types of environment can be gained from studies undertaken in other countries. A similar study of roe deer in Norway [39] did not identify a significant overlap of abomasal nematode fauna between roe deer and cattle, and concluded that roe deer pose no risk to domestic livestock. A study undertaken in Spain [40] concluded that there was significant cross-transmission of GI nematodes between wild deer (although they studied fallow and red deer) and cattle, but this was inferred only on the basis of eggs of generalist nematodes being present in high numbers in the wild deer population sampled. The strongest evidence of in-field cross-transmission of parasitic GI nematodes between wild (including roe deer) and domestic ruminants comes from Italy. Cerutti *et al.* [10] investigated nucleotide differences in mitochondrial and *ITS* ribosomal RNA genes of *H. contortus* in wild ruminants (including roe deer) and domestic sheep, and using molecular phylogenetic methods found that a single population of this parasite cycles between all hosts studied. The study in Italy corroborates the findings of this study. The inferences drawn from the statistical analysis of our data could be strengthened by similar molecular phylogenetic analysis to that undertaken by Cerutti *et al.* [10] on the specimens of *H. contortus* isolated from wild deer and further individuals of *H. contortus* isolated from sheep and cattle farms.

To confirm the potential of nematodes of roe deer to infect farmed ruminants, an *in vivo* cross-infection experiment with strains of nematodes isolated from wild roe deer was undertaken, rather than using nematode strains developed in the laboratory as has been used in previous studies of cross-infection between deer, cattle and sheep. This experiment confirmed that nematode populations isolated directly from wild roe deer successfully infected cattle and sheep. The main species that transmitted between all three host species was *H. contortus*, which has been shown before to be a generalist parasite able to infect all of these hosts [9]. This study also demonstrated in a direct cross-infection experiment that *O. leptospicularis* is a generalist parasite and can spread between roe deer, cattle and sheep. Indeed, previous studies report the presence of this parasite in wild fallow [41] and red deer [42], but do not demonstrate direct transmission to cattle. We also demonstrated in a direct cross-infection experiment that *S. spiculoptera* can pass from roe deer to cattle. Previous studies identified this species in cattle [43], but did not experimentally demonstrate direct transfer from wild deer. It is possible that the importance of other species pathogenic to cattle and sheep, such as *O. ostertagi* and *T. circumcincta*, were underestimated as the deer hunting season might not necessarily coincide with major periods of cross-transmission. Collection of further samples outside the hunting season would provide valuable data, and such samples could be obtained from individuals that die of natural causes or as road-kill.

The *in vivo* cross-infection experiment was carried out with the entire population of eggs recovered from roe deer faeces. As such, although high numbers of eggs were used, it is likely that only a small proportion of hatched nematodes were able to colonize, and in consequence a low level of infection was established. Out of the 17 nematodes recovered only three were *H. contortus*, and sequencing of these individuals for BZ resistance revealed that the anthelmintic-resistant *H. contortus* identified in roe deer were able to establish infection in the calf. This is a proof of concept and further conclusions regarding any fitness advantage or disadvantage in terms of colonization potential cannot be drawn owing to the low number of nematodes recovered and the use of a single experimental animal.

Because anthelmintic resistance is a serious and increasing problem in the UK in sheep, and a developing problem in cattle, this study sets out to assess whether deer can become infected with resistant nematodes from livestock. Infected deer could then potentially spread anthelmintic-resistant nematodes between farms. PCR isolation and sequencing of the *isotype-1 *β*-tubulin* of *H. contortus* isolated from wild roe deer identified BZ-resistant genotypes/alleles. The BZ-R status of this isolate was subsequently confirmed by *in vitro* tests. An EHT was undertaken on eggs extracted from faeces of the lamb infected with this isolate; 62.4% of the eggs hatched in 0.1 µg ml^−1^ thiabendazole, which is very similar to the 64% resistant allele frequency at codon 200 shown by sequencing of the β-tubulin gene of the *H. contortus* extracted directly from wild roe deer and to the 60% resistant allele frequency at codon 200 identified in *H. contortus* extracted from the experimentally infected lamb. These results are in agreement with suggestions that the 0.1 µg ml^−1^ thiabendazole can be used as an LD_99_ for BZ resistance in *H. contortus* [28].

Although the FECRT suggested the presence of BZ resistance, it was not demonstrated at a statistically significant level owing to a number of limitations of the study. First, the low number of animals used is below the recommended guideline of 10 [44], and this was due to constraints applied by the low number of larvae obtained from the artificial infection of the lamb used as amplification vessel. Second, accidental laceration of the abomasum of one lamb and loss of contents potentially affected the parasite count at slaughter and confounded the results. Third, the starting counts were much higher than the counts in the control or the ivermectin-treated groups, which disproportionately increased the chance of high post-treatment egg counts in the BZ-treated pair. A repeat FECRT with larvae collected from roe deer but designed according to the guidelines detailed by Coles *et al.* [44], and a minimum of 10 animals in each group, would be needed to establish the presence of phenotypic BZ resistance *in vivo*. Nevertheless, along with the supporting data from the *in vitro* tests, these results demonstrate the presence of BZ-resistant nematodes in untreated roe deer. No indication of resistance was obtained even to half-dose ivermectin, but, again, the low number of animals tested makes it difficult to draw firm conclusions.

The present findings should stimulate further larger-scale studies into the dynamics of cross-transmission of parasitic GI nematodes between wild deer and livestock. Given that in discontinuously grazed environments the timing of nematode transmission between wildlife and livestock is likely to be a predictable function of climate and habitat use [45], more detailed characterization of livestock–deer interaction through common use of pasture could provide the basis for recommendations to limit parasite transmission in both directions. If deer are sufficiently important as a vector of anthelmintic resistance, which is not yet proven, such principles could be incorporated into decision support systems for farmers. Of course, anthelmintic resistance can be brought onto farms by other means, especially with imported sheep, and, furthermore, it is possible that deer act as valuable natural refugia for drug-susceptible alleles, such that some exchange of parasites between domestic and wild ungulates is advantageous to the long-term sustainability of chemical parasite control on farms. Certainly, environmental change and increasing habitat fragmentation are altering patterns of contact at the wild–domestic interface, and disease control should take greater account of the whole ecosystem in a ‘one health’ approach [46]. Judging by parallel studies of infectious disease transmission between wildlife hosts [47], cross-sectional surveys will have limited power to infer whether deer act as transient hosts of parasites transmitted from livestock, or are important reservoir hosts in their own right. Further work is therefore needed before the epidemiological importance of parasite transmission between deer and livestock in different areas and situations can be specified, as well as whether and how this new knowledge should be incorporated into parasite control strategies.

In conclusion, wild roe deer have the potential to acquire benzimidazole-resistant *H. contortus* from cattle and sheep in the areas of intensive livestock farming, a process likely to be favoured by increasing deer populations and landscape fragmentation. Onward spread of anthelmintic-resistant nematodes to livestock by wild deer has the potential to be a serious issue, especially if this promotes dissemination of resistance between farms. However, this has not been proven in this study, and further research is necessary to elucidate the extent of cross-infection and its implications.

## Supplementary Material

Samples collected from wild deer

## Supplementary Material

Abomasal nematodes identified in wild roe deer

## Supplementary Material

Egg Hatch Test on eggs of H. contortus isolated from wild roe deer

## Supplementary Material

Faecal Egg Count Reduction Test (FECRT) for benzimidazole and ivermectin

## Notes

Ethical approval was not required for these samples as the deer were part of the national culling quota (undertaken by the Forestry Commission, UK) and not purposely slaughtered for this study. Consent for sample collection was obtained from the Forestry Commission Officers at Haldon Forest (Exeter), Ludlow (West Midlands) and the New Forest. Consent for sample collection from private grounds was obtained from the owners of the grounds, and samples were taken by the ground management staff. The lamb and calf experiments were contracted to Ridgeway Research, UK, and they were conducted in accordance to the Home Office regulations under the project licence PPL/30/2440 and study number RRSH-075-11-09. The experiment was screened by Ridgeway's internal ethical approval system.

## References

[1] Bohlender RE 1988 Economics of deworming beef cattle. Vet. Parasitol. 27, 67–71. (10.1016/0304-4017(88)90062-3) 3363844

[2] Stuedemann JA Ciordia H Myers GH McCampbell HC 1989 Effect of a single strategically timed dose of fenbendazole on cow and calf performance. Vet. Parasitol. 34, 77–86. (10.1016/0304-4017(89)90167-2) 2588472

[3] Williams JC Bechtol DT Hollis LC Herschler RC 1991 Effects of oxfendazole, levamisole, and ivermectin treatment on removal of inhibited *Ostertagia ostertagi* larvae and production parameters in feedlot steers. Agri-Practice 12, 14–20.

[4] Gross SJ Ryan WG Ploeger HW 1999 Anthelmintic treatment of dairy cows and its effect on milk production. Vet. Rec. 144, 581–587. (10.1136/vr.144.21.581) 10378289

[5] Charlier J Camuset P Claerebout E Courtay B Vercruysse J 2007 A longitudinal survey of anti-*Ostertagia ostertagi* antibody levels in individual and bulk tank milk in two dairy herds in Normandy. Res. Vet. Sci. 83, 194–197. (10.1016/j.rvsc.2006.12.005) 17258252

[6] Michel JF Richards M Altman JF Mulholland JR Gould CM Armour J 1982 Effect of anthelmintic treatment on the milk yield of dairy cows in England, Scotland and Wales. Vet. Rec. 111, 546–550. 7164331

[7] Nieuwhof GJ Bishop SC 2005 Costs of the major endemic diseases of sheep in Great Britain and the potential benefits of reduction in disease impact. Anim. Sci. 81, 23–29. (10.1079/ASC41010023)

[8] Hoberg EP Lichtenfels JR 1994 Phylogenetic systematic analysis of the *Trichostrongylidae* (*Nematoda*), with an initial assessment of coevolution and biogeography. J. Parasitol. 80, 976–996. (10.2307/3283448) 7799171

[9] Mcghee MB Nettles VF Rollor EA Prestwood AK Davidson WR 1981 Studies on cross-transmission and pathogenicity of *Haemonchus contortus* in white-tailed deer, domestic cattle and sheep. J. Wildl. Dis. 17, 353–364. (10.7589/0090-3558-17.3.353) 7310944

[10] Cerutti MC Citterio CV Bazzocchi C Epis S D'Amelio S Ferrari N Lanfranchi P 2010 Genetic variability of *Haemonchus contortus* (Nematoda: Trichostrongyloidea) in alpine ruminant host species. J. Helminthol. 84, 276–283. (10.1017/S0022149X09990587) 19889245

[11] Pato J 2009 Gastrointestinal nematode species shared by roe deer (*Capreolus capreolus*) and grazing cattle from Galicia. Asociación Interprofesional para el Desarrollo Agrario 1, 176–178.

[12] Pato FJ 2013 Gastrointestinal nematode infections in roe deer (*Capreolus capreolus*) from the NW of the Iberian peninsula: assessment of some risk factors. Vet. Parasitol. 196, 136–142. (10.1016/j.vetpar.2013.01.027) 23433640

[13] Coles GC 1997 Nematode control practices and anthelmintic resistance on British sheep farms. Vet. Rec. 141, 91–93. (10.1136/vr.141.4.91) 9265708

[14] Waghorn TS Leathwick DM Rhodes AP Lawrence KE Jackson R Pomroy WE West DM Moffat JR 2006 Prevalence of anthelmintic resistance on sheep farms in New Zealand. New Zealand Vet. J. 54, 271–277. (10.1080/00480169.2006.36710) 17151724

[15] Waghorn TS Leathwick DM Rhodes AP Jackson R Pomroy WE West DM Moffat JR 2006 Prevalence of anthelmintic resistance on 62 beef cattle farm in the North Island of New Zealand. New Zealand Vet. J. 54, 278–282. (10.1080/00480169.2006.36711) 17151725

[16] Sargison ND Jackson F Bartley DJ Moir ACP 2005 Failure of moxidectin to control benzimidazole-, levamisole- and ivermectin-resistant *Teladorsagia circumcincta* in a sheep flock. Vet. Rec. 156, 105–109. 15704550 10.1136/vr.156.4.105

[17] Blake N Coles G 2007 Flock cull due to anthelmintic-resistant nematodes. Vet. Rec. 161, 36 (10.1136/vr.161.1.36-b) 17617547

[18] Cringoli G 2006 FLOTAC, a novel apparatus for a multivalent faecal egg count technique. Parassitologia 48, 381–384. 17176947

[19] Bauer BU Pomroy WE Gueydon J Gannac S Scott I Pfister K 2010 Comparison of the FLOTAC technique with the McMaster method and the Baermann technique to determine counts of *Dictyocaulus eckerti* L1 and strongylid eggs in faeces of red deer (*Cervus elaphus*). Parasitol. Res. 107, 555–560. (10.1007/s00436-010-1893-z) 20502918

[20] Efron B Tibshirani R 1993 An introduction to the bootstrap*.* New York, NY: Chapman and Hall.

[21] Ministry of Agriculture, Fisheries and Food (MAFF). 1986 Manual of veterinary parasitological techniques. London, UK: HM Stationery Office.

[22] Skrjiabin KI Shikhobalova NP Shults RS 1956 Essentials of nematodology. Vol III – Trichostrongylids of animals and man. Moscow, Russia: Academy of Sciences of the USSR.

[23] Zarlenga DS Barry Chute M Gasbarre LC Boyd PC 2001 A multiplex PCR assay for differentiating economically important gastrointestinal nematodes of cattle. Vet. Parasitol. 97, 199–209. (10.1016/S0304-4017(01)00410-1) 11390072

[24] Zarlenga DS Hoberg EP Stringfellow F Lichtenfels JR 1998 Comparisons of two polymorphic species of *Ostertagia* and phylogenetic relationships within the Ostertagiinae (Nematoda: Trichostrongyloidea) inferred from ribosomal DNA repeat and mitochondrial DNA sequences. J. Parasitol. 84, 806–812. (10.2307/3284592) 9714215

[25] Helse M Christian E Schneider T 1999 Differences in the second internal transcribed spacer (ITS2) of eight species of gastrointestinal nematodes in ruminants. J. Parasitol. 85, 431–435. (10.2307/3285774) 10386433

[26] Coles GC Jackson F Pomroy WE Prichard RK von Samson-Himmelstjerna G Silvestre A Taylor MA Vercruysse J 2006 The detection of anthelmintic resistance in nematodes of veterinary importance. Vet. Parasitol. 136, 167–185. (10.1016/j.vetpar.2005.11.019) 16427201

[27] von Samson-Himmelstjerna G 2009 Standardization of the egg hatch test for the detection of benzimidazole resistance in parasitic nematodes. Parasitol. Res. 105, 825–834. (10.1007/s00436-009-1466-1) 19452165

[28] Cudekova P Varady M Dolinska M Konigova A 2010 Phenotypic and genotypic characterisation of benzimidazole susceptible and resistant isolates of *Haemonchus contortus*. Vet. Parasitol. 172, 155–159. (10.1016/j.vetpar.2010.04.022) 20684865

[29] Coles GC Rhodes AC Wolstenholme AJ 2005 Rapid selection for ivermectin resistance in *Haemonchus contortus*. Vet. Parasitol. 129, 345–347. (10.1016/j.vetpar.2005.02.002) 15845291

[30] Jacquiet P Cabaret J Cheikh D Thiam E 1997 Identification of *Haemonchus* species in domestic ruminants based on morphometrics of spicules. Parasitol. Res. 83, 82–86. (10.1007/s004360050213) 9000240

[31] Batty AF Chapmann DI 1970 Gastro-intestinal parasites of wild fallow deer (*Dama dama*). J. Helminthol. 44, 57–61. (10.1017/S0022149X0002143X)

[32] Batty AF Chapman DI Chapman N 1987 Prevalence of nematode parasites in wild fallow deer (*Dama dama*). Vet. Rec. 120, 599 (10.1136/vr.120.25.599-a) 3629863

[33] Morgan ER Milner-Gulland EJ Torgerson PR Medley GF 2004 Ruminating on complexity: macroparasites of wildlife and livestock. Trends Ecol. Evol. 19, 181–188. (10.1016/j.tree.2004.01.011) 16701252

[34] Morgan ER Lundervold M Medley GF Shaikenov BS Torgerson PR Milner-Gulland EJ 2006 Assessing risks of disease transmission between wild-life and livestock: the Saiga antelope as a case study. Biol. Conserv. 131, 244–254. (10.1016/j.biocon.2006.04.012)

[35] Maublanc ML Bideau E Picot D Rames JL Dubois M Ferte H Gerard JF 2009 Demographic crash associated with high parasite load in an experimental roe deer (*Capreolus capreolus*) population. Eur. J. Wildl. Res. 55, 621–625. (10.1007/s10344-009-0298-8)

[36] Morellet N Van Moorter B Cargnelutti B Angibault J-M Lourtet B Merlet J Ladet S Hewison AJM 2011 Landscape composition influences roe deer habitat selection at both home range and lanscape scales. Landsc. Ecol. 26, 999–1010. (10.1007/s10980-011-9624-0)

[37] Serrano Ferron E 2012 Digestive plasticity as a response to woodland fragmentation in roe deer. Ecol. Res. 1, 77–82. (10.1007/s11284-011-0872-x)

[38] Li S Hartemink N Speybroeck N Vanwambeke SO 2012 Consequences of landscape fragmentation for lyme disease risk: a cellular automata approach. PLoS ONE 7, e39612 (10.1371/journal.pone.0039612) 22761842 PMC3382467

[39] Kongsbak RH 2005 Gastrointestinal nematodes (*Nematoda: Trichostrongyloidea*) in cattle (*Bos taurus*), moose (*Alces alces*) and roe deer (*Capreolus capreolus*) in southern Norway. The effect of anthelmintic treatment in relation to a possible cross-infection. MSc thesis, University of Oslo http://urn.nb.no/URN:NBN:no-11934.

[40] Santin-Duran M Alunda JM Hoberg EP Concepcion de la F 2004 Abomasal parasites in wild sympatric cervids, red deer (*Cervus elaphus*) and fallow deer (*Dama dama*) from three localities across central and western Spain: relationship to host density and park management. J. Parasitol. 90, 1378–1386. (10.1645/GE-3376) 15715232

[41] Barth D Matzke P 1984 Gastro-intestinal nematodes of fallow deer (*Dama dama*) in Germany. Vet. Parasitol. 16, 173–176. (10.1016/0304-4017(84)90018-9) 6543050

[42] Suarez VH Busetti MR Fort MC Bedotti DO 1991 *Spiculopteragia spiculoptera*, *S. asymmetrica* and *Ostertagia leptospicularis* from *Cervus elaphus* in La Pampa, Argentina. Vet. Parasitol. 40, 165–168. 1763487 10.1016/0304-4017(91)90095-d

[43] Hinaidi HK Guttieres VC Supperer R 1972 The gastro-intestinal helminths of cattle in Austria. Zentralblatt Veterinarmedizin B. 19, 679–695. (10.1111/j.1439-0450.1972.tb00449.x) 4656080

[44] Coles GC Bauer C Borgsteede FHM Geerts S Klei TR Taylor MA Waller PJ 1992 World association for the advancement of parasitology (WAAVP) methods for the detection of anthelmintic resistance in nematodes of veterinary importance. Vet. Parasitol. 44, 35–44. (10.1016/0304-4017(92)90141-U) 1441190

[45] Morgan ER Medley G Torgerson PR Shaikenov PR Milner-Gulland EJ 2007 Parasite transmission in a migratory mutiple host system. Ecol. Model. 200, 511–520. (10.1016/j.ecolmodel.2006.09.002)

[46] Zinsstag J Schelling E Waltner-Toews D Tanner M 2011 From ‘one medicine’ to ‘one health’ and systemic approaches to health and well-being. Prevent. Vet. Med. 101, 148–156. (10.1016/j.prevetmed.2010.07.003) PMC3145159 20832879

[47] Craft ME Volz E Packer C Meyers LA 2009 Distinguishing epidemic waves from disease spillover in a wildlife population. Proc. R. Soc. B. 276, 1777–1785. (10.1098/rspb.2008.1636) PMC2674485 19324800

