# Supplementary material for: Wild deer as potential vectors of anthelmintic-resistant abomasal nematodes between cattle and sheep farms
Source: Proc Biol Sci. 2014 Apr 7;281(1780):20132985. doi: 10.1098/rspb.2013.2985 (PMC4027391; doi:10.1098/rspb.2013.2985)
Supplement: Samples collected from wild deer [file rspb20132985supp1.docx]

| **Site number** | **Site location** | **Species of deer collected** | **Number of deer collected (*)** | **Type of grazing environment** |
| --- | --- | --- | --- | --- |
| **Site 1** | Chepstow, Gloucestershire, West (W) England | Fallow | 5 | Farmed deer |
| **Site 2** | Bristol, South West (SW) England | Fallow | 5 | Farmed deer |
|  |  | Red | 5 |  |
| **Site 3** | North Somerset, W England | Red | 3 | Farmed deer |
|  |  | Roe | 5 | Wild deer grazing in areas of intensive livestock farming |
| **Site 4** | New Forest, South (S) England | Fallow | 4 | Wild deer grazing in areas of extensive cattle farming |
|  |  | Red | 3 |  |
| **Site 5** | Exeter, SW England | Fallow | 5 | Wild deer grazing in areas of intensive livestock farming |
|  |  | Red | 3 |  |
| **Site 6** | Taunton, SW England | Roe | 5 | Wild deer grazing in areas of intensive livestock farming |
| **Site 7** | Ludlow, W England | Fallow | 5 | Wild deer grazing in areas of intensive livestock farming |
