# Supplementary material for: Wild deer as potential vectors of anthelmintic-resistant abomasal nematodes between cattle and sheep farms
Source: Proc Biol Sci. 2014 Apr 7;281(1780):20132985. doi: 10.1098/rspb.2013.2985 (PMC4027391; doi:10.1098/rspb.2013.2985)
Supplement: Egg Hatch Test on eggs of H. contortus isolated from wild roe deer [file rspb20132985supp3.docx]

| **Concentration µg/ml** | **First repeat** | | **Second repeat** | | **Third repeat** | |
| --- | --- | --- | --- | --- | --- | --- |
|  | **Total number of eggs** | **Number of unhatched eggs** | **Total number of eggs** | **Number of unhatched eggs** | **Total number of eggs** | **Number of unhatched eggs** |
| **0 (control)** | 117 | 0 | 134 | 0 | 136 | 1 |
| **0.01** | 95 | 1 | 135 | 0 | 184 | 4 |
| **0.025** | 137 | 2 | 138 | 5 | 184 | 4 |
| **0.05** | 132 | 30 | 135 | 26 | 152 | 35 |
| **0.1** | 108 | 36 | 92 | 32 | 182 | 81 |
| **0.185** | 120 | 77 | 130 | 87 | 148 | 103 |
| **0.25** | 150 | 113 | 149 | 140 | 172 | 142 |
| **0.3** | 124 | 117 | 163 | 158 | 132 | 127 |
