# Supplementary material for: Wild deer as potential vectors of anthelmintic-resistant abomasal nematodes between cattle and sheep farms
Source: Proc Biol Sci. 2014 Apr 7;281(1780):20132985. doi: 10.1098/rspb.2013.2985 (PMC4027391; doi:10.1098/rspb.2013.2985)
Supplement: Faecal Egg Count Reduction Test (FECRT) for benzimidazole and ivermectin [file rspb20132985supp4.docx]

| **Treatment group** | **Individual** | **FEC pre-treatment** | **FEC post-treatment** | **Efficacy of treatment (%)** | **Abomasal nematodes** |
| --- | --- | --- | --- | --- | --- |
| **Control** | 1 | 1850 | 2800 | N/A | 615 |
|  | 2 | 2750 | 2150 | N/A | 225 |
| **Fenbendazole (5mg/kg)** | 1 | 12150 | 1400 | 88.5 | 35* |
|  | 2 | 7650 | 400 | 94.7 | 273 |
| **Ivermectin (0.1mg/kg)** | 1 | 2950 | 0 | 100 | 0 |
|  | 2 | 2150 | 0 | 100 | 0 |

* At post-mortem, the abomasum was cut open accidentally and approximately half of the abomasal contents were lost. This may have resulted in underestimating the *H. contortus* burden in this animal.
